# Toothache

**Published:** 1899-11

**Authors:** M. G. Price

**Affiliations:** Mosheim, Tenn.


### Toothache.

*Editor Medical Summary:*—We have been experimenting with acetanilid in a rebellious tooth of our own. Pack the cavity with dry acenatilid and in a few minutes there is no toothache. A more convenient way is a saturated solution of alcohol and acetanilid with a few drops of oil caryophyllus. This preparation is an elegant one and meets many a demand, but a paste made of the oil just mentioned and arsenous acid, while it is painful for a few minutes, will soon leave no nerve to ache if applied on a little cotton.

M. G. Price, M.D.

Mosheim, Tenn.

